# Integrated 16S rDNA Gene Sequencing and Untargeted Metabolomics Analyses to Investigate the Gut Microbial Composition and Plasma Metabolic Phenotype in Calves With Dampness-Heat Diarrhea

**DOI:** 10.3389/fvets.2022.703051

**Published:** 2022-02-15

**Authors:** Zunxiang Yan, Kang Zhang, Kai Zhang, Guibo Wang, Lei Wang, Jingyan Zhang, Zhengying Qiu, Zhiting Guo, Xiaoping Song, Jianxi Li

**Affiliations:** ^1^Engineering and Technology Research Center of Traditional Chinese Veterinary Medicine of Gansu Province, Lanzhou Institute of Husbandry and Pharmaceutical Sciences, Chinese Academy of Agricultural Sciences (CAAS), Lanzhou, China; ^2^College of Veterinary Medicine, Northwest A&F University, Yangling, China

**Keywords:** dampness-heat diarrhea, gut microbiome, metabolomics, 16S rDNA, pathogenesis

## Abstract

Dampness-heat diarrhea (DHD), a common syndrome in Chinese dairy farms, is mainly resulted from digestive system disorders, and accompanied with metabolic disorders in some cases. However, the underlying mechanisms in the intestinal microbiome and plasma metabolome in calves with DHD remain unclear. In order to investigate the pathogenesis of DHD in calves, multi-omics techniques including the 16S rDNA gene sequencing and metabolomics were used to analyze gut microbial compositions and plasma metabolic changes in calves. The results indicated that DHD had a significant effect on the intestinal microbial compositions in calves, which was confirmed by changes in microbial population and distribution. A total of 14 genera were changed, including *Escherichia-Shigella, Bacteroides*, and *Fournierella*, in calves with DHD (*P* < 0.05). Functional analysis based on the Kyoto Encyclopedia of Genes and Genomes (KEGG) annotations indicated that 11 metabolic functions (level 2) were significantly enriched in DHD cases. The untargeted metabolomics analysis showed that 440 metabolites including bilineurin, phosphatidylcholine, and glutamate were significantly different between two groups (VIP > 1 and *P* < 0.05), and they were related to 67 signal pathways. Eight signal pathways including alpha-linolenic acid, linoleic acid, and glycerophospholipid metabolism were significantly enriched (*P* < 0.05), which may be potential biomarkers of plasma in calves with DHD. Further, 107 pairs of intestinal microbiota-plasma metabolite correlations were determined, e.g., *Escherichia-Shigella* was significantly associated with changes of sulfamethazine, butyrylcarnitine, and 14 other metabolites, which reflected that metabolic activity was influenced by the microbiome. These microbiota-metabolite pairs might have a relationship with DHD in calves. In conclusion, the findings revealed that DHD had effect on intestinal microbial compositions and plasma metabolome in calves, and the altered metabolic pathways and microorganisms might serve as diagnostic markers and potential therapeutic targets for DHD in calves.

## Introduction

Diarrhea, the most common disease in calves, is one of the major causes of economic loss in cattle herds worldwide (1, 2). Neonatal calves are susceptible to bovine coronavirus, *Escherichia coli*, bovine rotavirus, and *Cryptosporidium parvum*, which can cause diarrhea in calf (3–5). In addition, some non-infectious factors including colostrum management, calf housing, and hygiene are inducer of diarrhea in calf (6, 7). The rational use of traditional Chinese medicine (TCM) to treat this disease has certain advantages (8). Different types of diarrhea require different prescriptions based on the theory of TCM. According to the clinical symptoms, diarrhea can be divided into dampness-heat diarrhea (DHD), spleen deficiency diarrhea, and kidney deficiency diarrhea (9–11). DHD is one of the most common syndromes in Chinese cattle herds. According to traditional Chinese veterinary medicine (TCVM), DHD is usually caused by the exogenous heat and dampness toxin pathogens invading the intestine (12). The main clinical symptoms of DHD in calves are hyperthermia, sticky and loose stools with blood and mucus, red tongue, and thick greasy tongue-coating (9, 13). The mechanism of DHD has not been fully elucidated. This study provides new insights on understanding the pathogenesis and rational treatment of DHD in calves.

The alteration of gut microbial composition is associated with many diseases (14). Diarrheal diseases often alter the richness and diversity of intestinal flora (15, 16). Therefore, the microbiota is an important factor in the maintenance of health and the development of disease (17). The 16S rDNA gene amplicon sequencing, a powerful tool for routine microbial identification, can help researchers to identify, categorize, and understand the complex interactions between the host, pathogen, and microbiome (18, 19). Studies have confirmed that the increase of *Firmicutes* and *Bacteroidetes* proportion is an important manifestation of diarrhea, and diarrhea could be alleviated when this abnormal proportion was adjusted to be normal (20, 21). Gegenqinlian decoction, a classical TCM prescription, regulated the balance of intestinal mucosa flora in mice with diarrhea induced by high temperature and humidity (22). Therefore, it might be a helpful strategy for elucidation of the pathogenic mechanism of DHD to study of gut microbial composition in calves.

Qualitative and quantitative analysis of low molecular weight metabolites in biological samples can reflect the influence of diseases on metabolic in body (23, 24). In recent years, a great deal of research has been conducted on the metabolomics in a lot of diseases (25–28). The 16S rRNA gene sequencing with untargeted metabolomics can present much better perspectives on physiological and metabolic mechanisms involved in the pathogenesis of DHD. Some metabolites in plasma such as chenodeoxycholic acid and creatinine were found to the biomarkers for colorectal cancer diagnosis and prognosis (29). Metabolomics was utilized to study the molecular mechanisms of Atractylodis Rhizoma in the treatment of spleen deficiency (30). Therefore, we hypothesized that the changes of metabolic and intestinal microbiological might be related to DHD in calves. In this study, the difference in fecal microbiome and plasma metabolic profile between DHD calves and healthy calves was detected by 16S ribosomal DNA gene sequencing and metabolomics, and the results revealed that DHD had effect on intestinal microbial compositions and plasma metabolome, and the altered metabolic pathways and microorganisms might serve as diagnostic markers and potential therapeutic targets for DHD in calves.

## Materials and Methods

### Experimental Animals

The experiment was carried out in a dairy farm in north-western China. The calves in this study were similar in genetic background and age, and all of enrolled animals were housed and fed under same conditions. A total of 6 DHD calves and 6 healthy calves were enrolled in this study. The standards of dampness-heat diarrhea in the literature are mostly determined by primary and secondary symptoms (9, 10, 13). The inclusion criteria for DHD cases were required two main symptoms and secondary symptoms, or three main symptoms (Table 1). The specific clinical syndrome differentiation was carried out by the author and a Chinese veterinary expert. According to the diagnostic criteria, we screened 6 eligible calves from 129 diarrhea cases as DHD group and six healthy calves as the control group. The enrolled calves were not given any probiotics or antibiotics prior to sample collection. All animal procedures were carried out in accordance with the Guidelines on Laboratory Animal Ethics Commission of the Lanzhou Institute of Husbandry and Pharmaceutical Sciences of CAAS (SYXK [Gan] 2019-0002).

**Table 1:** Diagnostic criteria for DHD calves.

Main symptoms	Diarrhea, mucus or bloody purulent stool, red tongue, thick greasy tongue-coating
Secondary symptoms	Hyperthermia, shortness of urination, abdominal pain, anal burning loose stools like water, tenesmus, dry nose, thirst and small amount

### Fecal Sample Collection and DNA Extraction

Fresh fecal samples were collected by inserting an anal swab into the anus. The anal swabs were deposited into a sterile sampling tube and sent to the laboratory within 2 h. The fecal samples were immediately frozen in liquid nitrogen until the microbial DNA was extracted.

Microbial DNA was extracted from each fecal sample using the HiPure Fecal DNA Kit (Magen, Guangzhou, China) according to kit instructions. The DNA purity was determined using a Nanodrop microspectrophotometer (NanoDrop 2000, Thermo Fisher Scientific, America) and DNA integrity was investigated using the agarose gel electrophoresis.

### Plasma Sample Collection and Metabolite Extraction

Blood was collected from the jugular vein of the calves and stored in an anticoagulant tube containing ethylenediaminetetraacetic acid (EDTA), and was temporarily stored in an incubator at 4°C and sent to the laboratory within 2 h. The plasma was separated after centrifugation at 3,000 rpm, and 4°C for 15 min, and then frozen at −80°C until metabolite extraction.

Metabolites were extracted separately from plasma. After addition of 300 μL of methanol and 20 μL internal references in 100 μL plasma, the samples were mixed briefly and sonicated for 5 min in an ice-water bath. Following incubation at −20°C for 2 h, the mixture was centrifuged at 13,000 rpm at 4°C for 15 min. The supernatants were transferred to liquid chromatography-mass spectrometry (LC-MS) vials and stored at −80°C until the UHPLC-QE Orbitrap/MS analysis (Agilent, America).

### 16S RDNA Gene Sequencing Analysis

The 16S rDNA V3-V4 region was amplified using a specific primer with barcode 341F: CCTACGGGNGGCWGCAG; 806R: GGACTACHVGGGTATCTAAT (31). The prokaryotic 16S fragment was amplified by a relevant PCR reagent (Toyobo, Japan), and the PCR conditions consisted of an initial denaturation at 94°C for 2 min, followed by 30 cycles at 98°C for 10 s, 62°C for 30 s, and 68°C for 30 s and a final extension at 68°C for 5 min. PCR reactions were performed in triplicate.

The amplification material was extracted from a 2% agarose gel and purified using the AxyPrep DNA gel extraction kit (Axygen Biosciences, Union City, CA, USA) according to the manufacturer's instructions, and quantified using the ABI Step-OnePlus Real-Time PCR System (Life Technologies, Foster City, USA). The purified amplicons were polymerized on the Illumina platform with equimolar and paired-end sequence (PE250) according to the standard protocol. The original reads were stored in the NCBI Sequence Read Archive database (PRJNA713974).

DNA library sequencing was performed by Genedenovo Biotechnology Co., Ltd (Guangzhou, China) on the Illumina HiseqTM 2500. To obtain the effective tags, the raw tags of sequences were spliced and filtered. Detailed steps are described in the Supplementary Material. The effective tags were clustered into operational taxonomic units (OTUs) with >97% similarity using UPARSE (32). The representative sequences were classified by a naive Bayesian model with an RDP classifier (version 2.2) based on the SILVA database (version 132) with a confidence threshold of 0.8. The abundance statistics of taxonomy were visualized using KRONA (version 2.6) (33–35). The beta-diversity was assessed by calculating the weighted unifrac distances matrix and Bray-Curtis distance matrix dissimilarity, which was visualized through the use of principal coordinate analysis (PCoA). Species comparison among groups of various classification levels was calculated by the Wilcoxon rank test (*P* < 0.05). Tax4Fun functional prediction was used to infer the Kyoto Encyclopedia of Genes and Genomes (KEGG) pathway analysis of the OTUs. Analysis of function difference between groups was calculated by the Wilcoxon rank test.

### Metabolomics Analysis

The extracted metabolites were detected and quantitated with a liquid chromatography-tandem mass spectrometry (LC-MS/MS). Details of LC-MS/MS analysis were available in the Supplementary Material.

A data matrix containing retention time (RT), mass/charge ratio (M/Z), and peak strength was generated by preprocessing the MS raw data using the profile analysis software. Principal component analysis (PCA) and orthogonal partial least squares discriminant analysis (OPLS-DA) algorithms were used to compare the metabolite distribution using R package models (http://www.r-project.org/). The variable importance in projection (VIP) values of OPLS-DA and the *P*-values of the *t*-test were used to screen the metabolites with significant differences between the two groups (36). Those with a *P* < 0.05 on the *t*-test and VIP > 1 were preliminarily determined as the different metabolites between two groups. Pathway analysis and enrichment analysis of the metabolites were carried out by locating them to the associated pathway. The calculated *P*-value was corrected by false discovery rate (FDR), and FDR ≤ 0.05 as the threshold. The pathway satisfied this condition was defined as the pathway of significant enrichment in differential metabolites.

### Correlation Analysis of Fecal Microbiota and Plasma Metabolites

Bidirectional orthogonal projections to latent structures (O2PLS) analysis were used to integrate the microbiota and metabolomic data (37, 38). The significantly different metabolites and microbes were calculated by the Pearson statistical method, and the final correlation and the network map between them were obtained (39).

## Results

### Differences in Bacterial Communities Between DHD Calves and Healthy Calves

**Figure 1:**
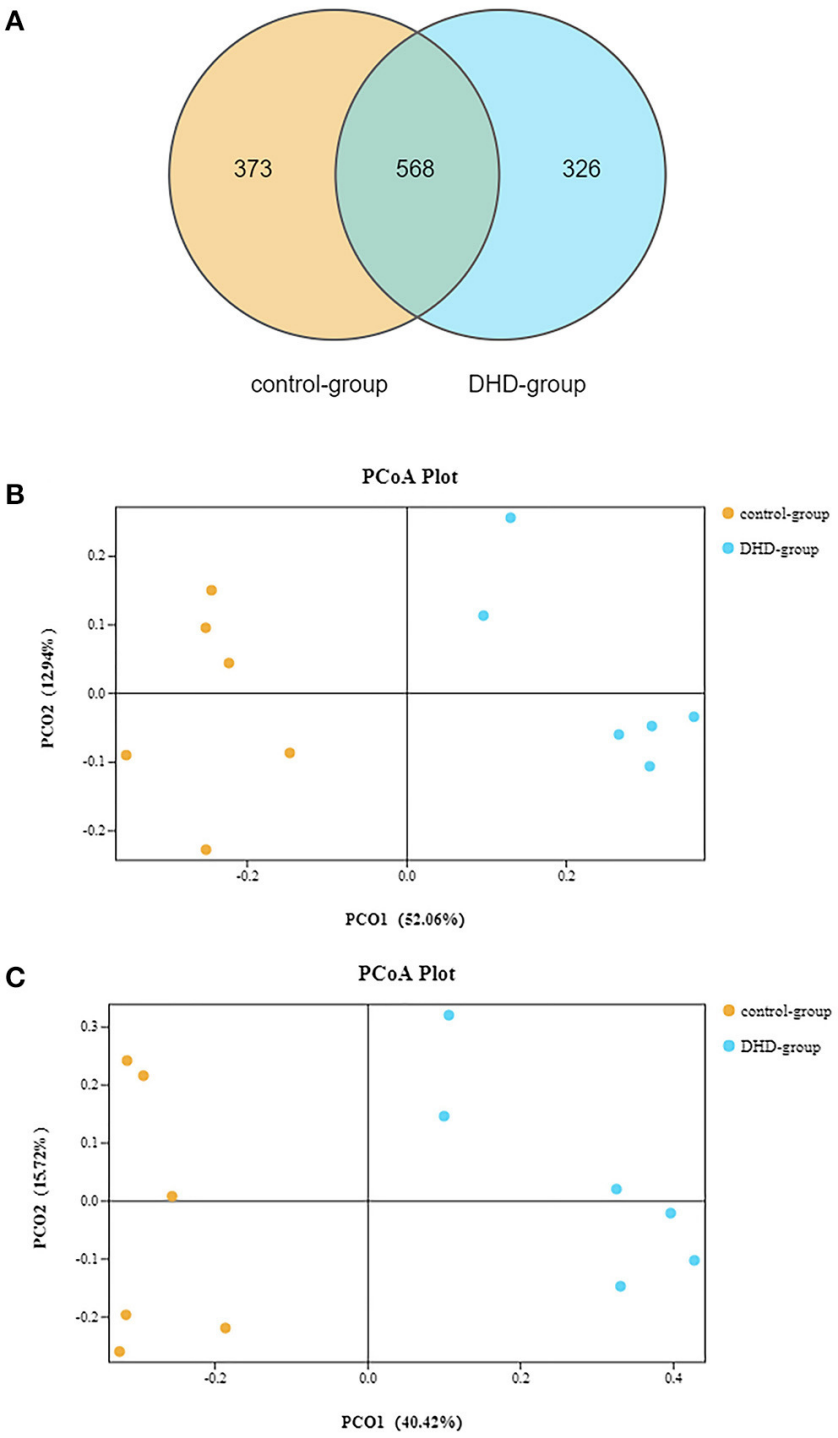
Gut microbial diversity in DHD and healthy calves. **(A)** Venn diagram showing the number of common or unique OTUs. **(B)** PCA by the weighted Unifrac of beta diversity of the OTU levels. **(C)** PCA by Bray-Curtis analysis of beta diversity at the OTU levels.

The fecal microbiome diversity of calves with DHD was investigated by 16S rDNA gene sequencing. The similarity of sequences contained in the same OTUs was ≥97%. The total number of effective tags and OTUs is shown in Table 2, OTUs was over 1,000 and effective tags was between 14,338 and 42,083 in samples. The Venn diagram (Figure 1A) shows the common and unique OTUs, 568 was the common OTUs, 373 OTUs in the control group, and 326 OTUs in DHD group. In order to characterize the similarity and difference in community abundance and composition, the beta diversity of microbiota was measured by PCoA. The result of the weighted Unifrac distance was ANOSIM *R* = 0.907, *P* = 0.002 (Figure 1B), and the Bray-Curtis analysis was ANOSIM *R* = 0.876, *P* = 0.004 (Figure 1C), both analyses indicated that the microflora of DHD group was significantly different from that of the control group.

**Table 2:** Tags and OTU quantity statistics in DHD and healthy calves.

**Samples**	**Total tags**	**Unique tags**	**Taxon tags**	**Unclassified tags**	**Singleton tags**	**OTUs**
Control 1	76563	43986	62225	0	14338	1043
Control 2	85298	49245	62079	0	23219	1027
Control 3	82507	45709	57577	0	24930	1151
Control 4	79651	49293	58272	0	21379	1291
Control 5	76206	50334	59974	0	16232	1077
Control 6	75641	48083	58190	0	17451	1212
DHD 1	81537	39910	58959	0	22578	1092
DHD 2	84150	35619	49722	0	34428	1074
DHD 3	79921	38741	52500	0	27421	1144
DHD 4	83273	44002	59353	0	23920	1153
DHD 5	91954	36162	68664	0	23290	1114
DHD 6	93275	35938	51192	0	42083	1038

### Alterations in Intestinal Microbiota Composition in Calves With DHD

**Figure 2:**
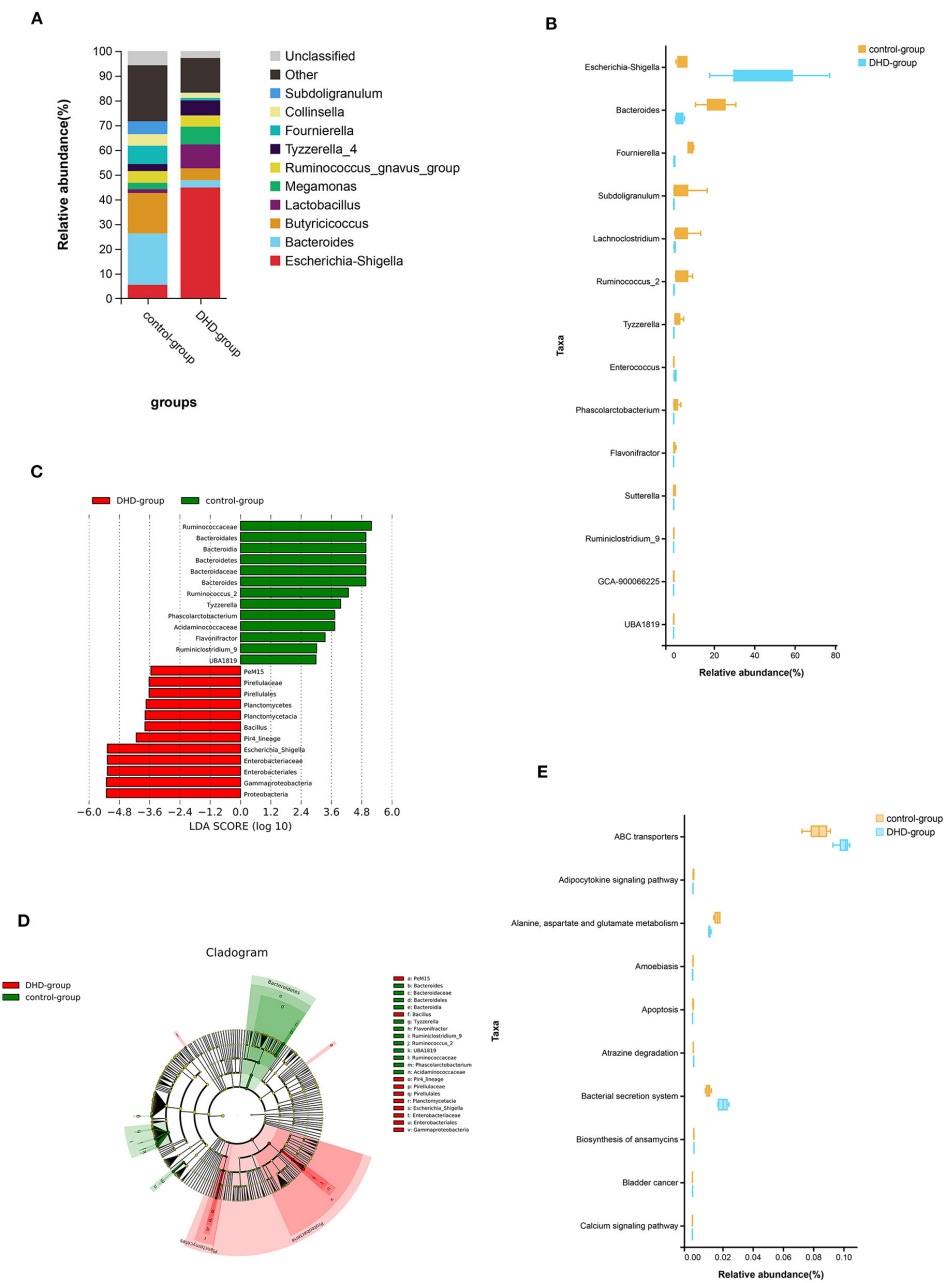
Fecal microbial abundance and diversity in DHD calves and healthy calves. **(A)** Taxonomic distributions of bacteria at the genus level (top 10) between DHD and healthy calves. **(B)** Box plot of the generic bacteria with significantly different between these two groups. Data for each group are shown as relative abundances. The Wilcoxon rank-sum test was used for statistical analysis. **(C)** The cladogram obtained by linear discriminant analysis effect size (LEfSe) analysis showed the phylogenetic distribution of the microflora of DHD calves and control calves from phylum to genus. **(D)** LDA score histograms used to identify bacterial genera (LDA score > 2) differed significantly between DHD calves and control calves. **(E)** Box plot of the top 10 significantly different KEGG pathways that were predicted between these two groups.

The relative abundance of the top 10 bacteria at the genus level is shown in Figure 2A, which indicates that the bacterial composition of feces in healthy and DHD calves is different, and *Escherichia-Shigella* (44.84%) was the predominant genus in the diseased calves. In healthy calves, the prevalent genera were *Bacteroides* (20.72%), *Fournierella* (7.38%), and *Escherichia-Shigella* (5.50%). The Wilcoxon rank sum test was used to compare the fecal bacterial community at the genus level, and 14 genera were significantly different between the two groups (*P* < 0.05). In these discriminatory genera, the abundance of *Escherichia-Shigella* and *Enterococcus* were increased significantly, and the abundance of *Bacteroides, Fournierella, Subdoligranulum, Lachnoclostridium*, and other 8 genera were decreased significantly in DHD calves (Figure 2B).

Linear discriminant analysis effect size (LEfSe) was used to further determine whether there was a differential enrichment of specific bacterial groups in calves. A total of 25 discriminatory taxa were clearly displayed in the linear discriminant analysis (LDA) score cutoff of 2.0 (Figure 2C). The *Pir4_lineage, Bacillus*, and *Escherichia_Shigella* were significantly over-represented in feces of DHD calves, whereas *Flavonifractor, Tyzzerella, Ruminiclostridium_9, Phascolarctobacterium, UBA1819, Bacteroides*, and *Ruminococcus_2* were the crucial microorganisms in healthy calves. A cladogram of the taxonomic hierarchy of fecal microbiota from phylum to genus illustrated a remarkable difference in the phylogenetic distribution of the microbiota between DHD calves and healthy calves (Figure 2D).

Tax4Fun was used to predict KEGG pathways in 16S rDNA sequencing result, and 284 pathways were predicted, most of which were related to metabolism (Supplementary Table 1), and 170 pathways were significantly different between DHD calves and healthy calves (*P* < 0.05). The top 10 pathways including adipocytokine and calcium, alanine, aspartate and glutamate metabolism, and apoptosis were shown in Figure 2E.

### Correlation Network Analysis

**Figure 3:**
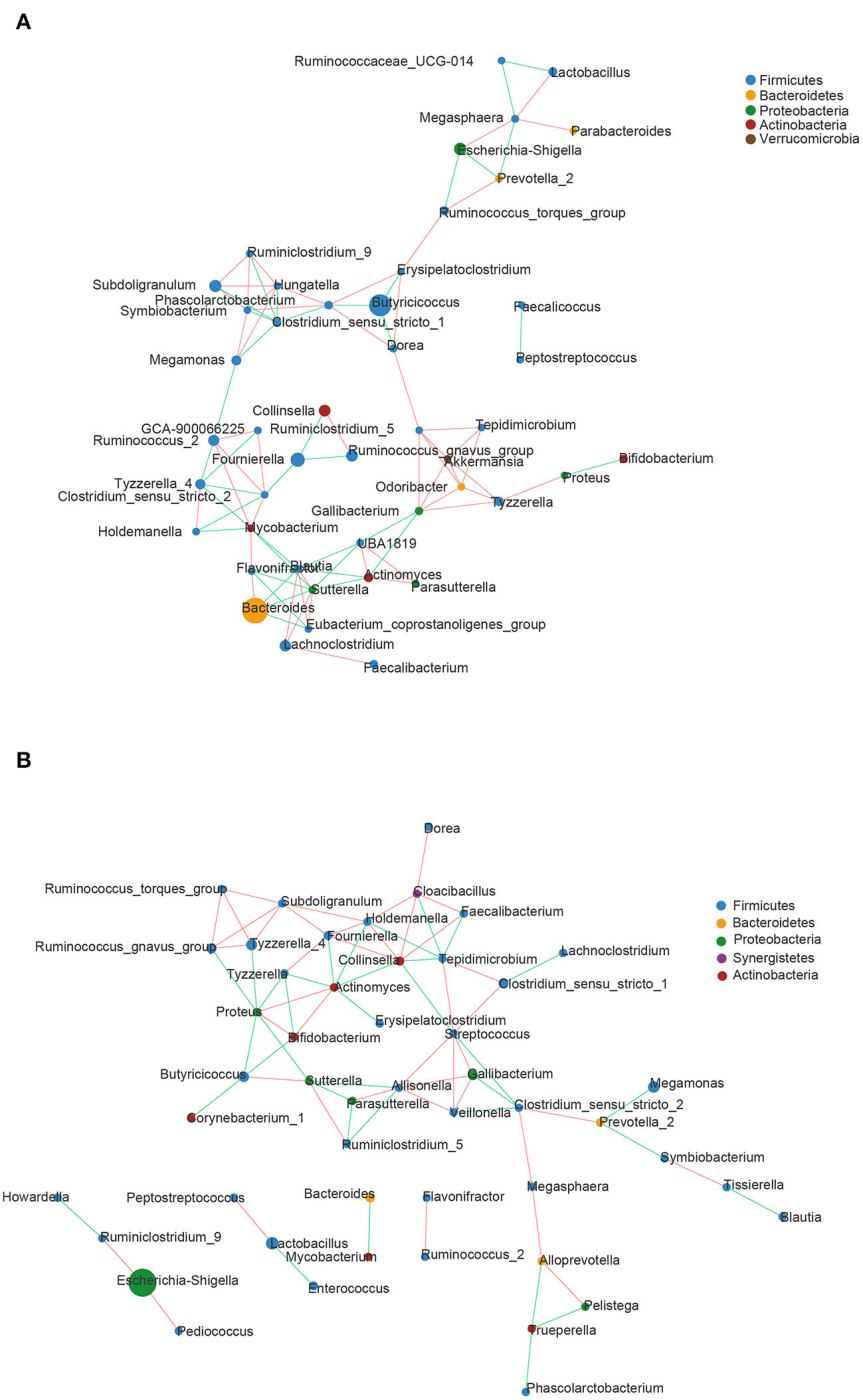
Correlation network diagrams of the 50 richest abundant genera of **(A)** healthy and **(B)** DHD calves. The lines between nodes represent the Spearman correlation and the color intensity represents the correlation coefficient (red, positive; green, negative). The color of the genera was based on phylum affiliation, and the size indicates average relative abundance.

Correlation network analysis was carried out at the genus level to determine whether DHD was associated with changes in the related structures and putative interactions of the intestinal microbiota. The correlation network diagrams of the top 50 genera with the richest abundant in DHD animals and healthy animals were represented in Figures 3A,B. The network had more edges (95 vs. 81) and higher mean degree (3.725 vs. 3) in the control group than those in the DHD group, which showed that the interaction structures of genus in the two groups were different.

### Metabolic Variation Analysis

**Figure 4:**
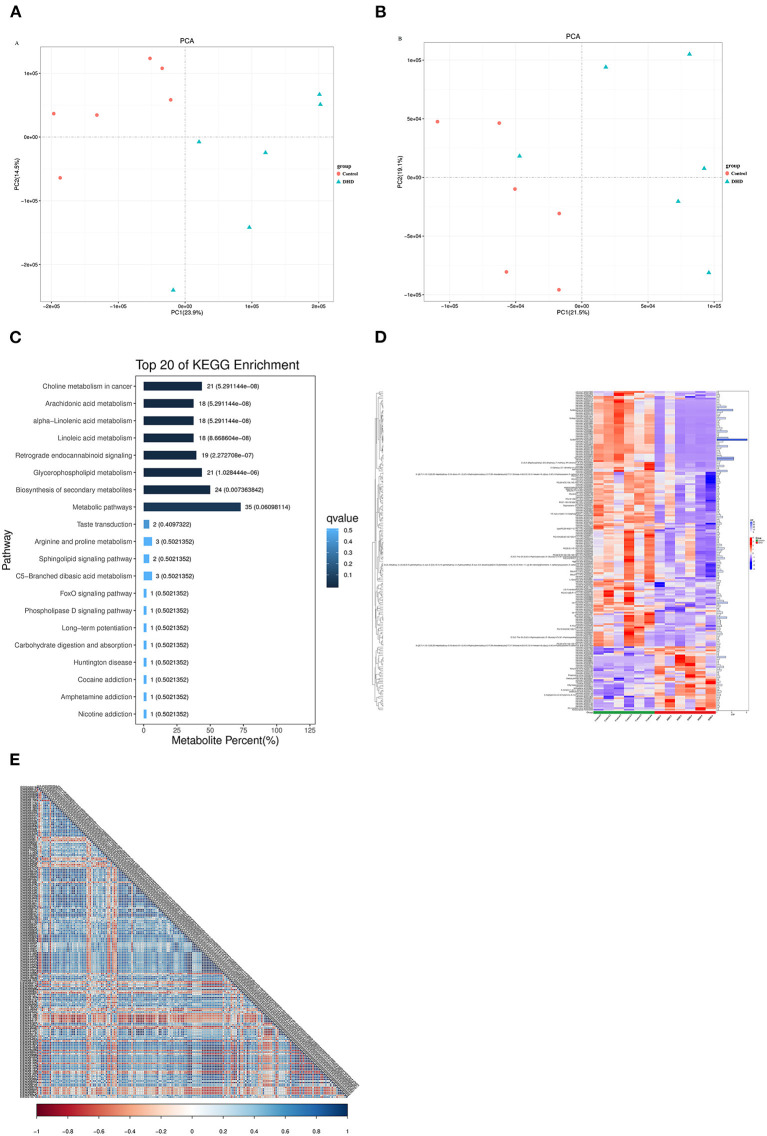
Plasma metabolic profiles in DHD and control calves. PCA score of plasma metabolite analysis between DHD calves and healthy calves in **(A)** positive ion mode and **(B)** negative ion mode. **(C)** Results of the KEGG pathway enrichment analysis of significantly different metabolites (top 20). The horizontal axis represents the percentage of the number of metabolites in this pathway to the total number of significantly different metabolites, and the value on the histogram is the number of differential metabolites in this pathway and the Q value. **(D)** Significant differences in metabolites between DHD calves and control calves are shown in the hierarchical clustering and heat map on the left (columns, individual; rows, specific metabolite). The histogram on the right represents the VIP score for each metabolite derived from the OPLS-DA model. **(E)** Correlation coefficient matrix thermograph illustrating the functional correlation between the significantly altered metabolites in plasma. The correlation coefficient is expressed by color, blue is positive correlation and red is negative correlation. The darker the color, the stronger the correlation.

Metabolite identification results showed that there were 8,145 and 7,074 peaks in positive and negative ion modes, respectively. PCA analysis indicated that the metabolites in plasma from tested calves were clearly separated in the positive and negative mode (Figures 4A,B). Potential metabolic biomarkers were screened using the multivariate model OPLS-DA (VIP > 1 and *P* < 0.05), and 440 potential metabolic biomarkers (Supplementary Table 2) were different between DHD calves and healthy calves.

To compare the main biochemical metabolic pathways and signal transduction pathways involved in differential metabolites between DHD calves and healthy calves, the enrichment analysis of pathways on these metabolites was evaluated by KEGG. The significantly different metabolites were enriched into 67 pathways, 8 pathways of them were significantly changed in DHD calves, including metabolism of arachidonic acid, alpha-linolenic acid, linoleic acid, and glycerophospholipid (*P* < 0.05, Figure 4C).

In present study, the metabolites with VIP > 2 were considered to be significantly different, and 194 metabolites were identified (Supplementary Table 3). The tendencies in variation of the 194 metabolites were depicted in a heat map (Figure 4D), and 39 metabolites were increased and 155 metabolites were decreased in DHD calves (*P* < 0.05). Most of different metabolites was Lipids and lipid-like molecules, and glycerophospholipids such as PC [16:0/20:2(11Z,14Z)], PC [20:2(11Z,14Z)/14:0], and PC [22:4(7Z,10Z,13Z,16Z)/16:0] were down-regulated in DHD calves, and some fatty acyls such as ethyl dodecanoate and butyrylcarnitine presented at higher levels in DHD calves. In addition, the correlation coefficient matrix thermograph showed an obvious correlation in these 194 metabolites (Figure 4E).

### Correlation Analysis of Intestinal Microbiota and Plasma Metabolites

**Figure 5:**
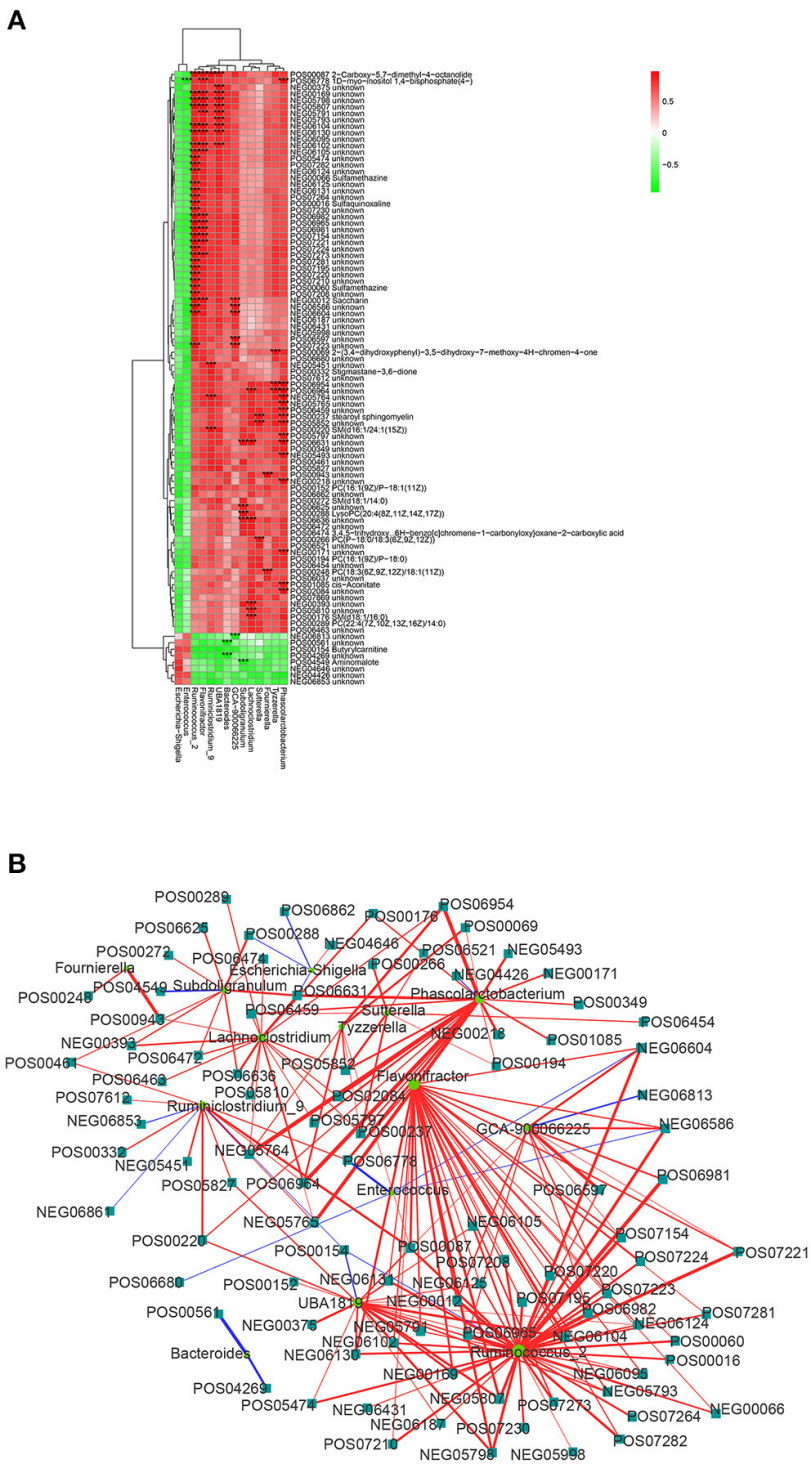
Relationship between fecal microorganisms and plasma metabolites. **(A)** Heat map summarizing the correlation between significantly different fecal microbiota and significantly altered metabolites in plasma (red, positive correlation; green, negative correlation). ***Indicates the significant microbiota-metabolite correlations (|cor| > 0.75 and *P* < 0.01). **(B)** Network diagram of significant microbiota-metabolite correlations. The circle represents the altered bacteria, and the rectangle represents the altered metabolites.

In order to investigate the functional correlation between 14 microbial communities at the genus level and 194 metabolites with significant differences, a correlation matrix was calculated using Spearman's correlation coefficient. As shown in Figure 5, 107 pairs of microbiota-metabolite were determined, including 102 positive correlations and 5 negative correlations (| cor | ≥ 0.75 and *P* < 0.01). Specially, *Ruminococcus_2, Phascolarctobacterium, Flavonifractor*, and *UBA1819* were significantly associated with 33, 15, 17, and 10 plasma metabolites, respectively. In addition, *Fournierella* was positively correlated with PC [18:3(6Z,9Z,12Z)/18:1(11Z)] and an unknown metabolite. *Subdoligranulum* was negatively correlated with aminomalote and positively correlated with LysoPC [20:4(8Z,11Z,14Z,17Z)]. *Tyzzerella* and *Enterococcus* were positively correlated with 3 metabolites and negatively correlated with 1 metabolite, respectively. The details of these significant microbiota-metabolite correlations were shown in Supplementary Table 4. Furthermore, the *Ruminococcus_2* node was the largest in fecal flora and the dominant bacteria in the network diagram. There were 33 metabolites with significant positive correlation with *Ruminococcus_2*, including saccharin, sulfamethazine and 2-carboxy-5,7-dimethyl-4-octanolide. In the significantly different metabolites, 2-carboxy-5,7-dimethyl-4-octanolide nodes were the largest in the network diagram, and with a positive correlation to *Ruminococcus_2, Flavonifractor, Ruminiclostridium_9* and *UBA1819* (Figure 5B). These data suggested that there is a significant taxonomic disturbance in the fecal microbiota in calves with DHD, which might lead to changes in metabolism.

## Discussion

Calf diarrhea is a growing concern worldwide because it has caused serious economic loss in cattle industry. Although DHD is a common type of diarrhea in calves, the mechanisms of this disease have not been fully elucidated. Previous studies revealed that intestinal microflora play a key role in maintaining metabolic balance (40). Most of metabolites in the blood were used to as biomarkers for diarrhea-related diseases (41). Thus, to study the fecal microbiota composition and plasma metabolomics characteristics of DHD calves may help to understand the pathogenesis of DHD and develop appropriate intervention strategies. This study is the first to characterize the fecal microbiome and plasma metabolites in calves with DHD by integrating 16S rDNA gene sequencing and LC–MS-based metabolomics approaches. The results revealed that the intestinal microbiota composition and plasma metabolic phenotype of DHD calves were significantly different from those of healthy calves.

As a complex ecosystem of the body, the intestinal microbiota is maintained a dynamic balance in healthy host. Many of disease were due to the disturbance of the intestinal microbiota (42, 43). In present study, 16S rDNA gene sequencing was performed to investigate the alteration of fecal microbiota in DHD. There were significant differences in the species abundance and composition of fecal microbes between DHD calves and healthy calves, which suggested that DHD may be related to the significant changes of the intestinal microbiota composition. Compared with healthy calves, the proportion of *Bacteroidetes* in calves with DHD was significantly down-regulated, but *Proteobacteria* was significantly up-regulated. *Proteobacteria* is the most abundant bacterial phylum in the intestinal microbiota of DHD calves, and it can produce lipopolysaccharide (LPS), which in turn induces inflammation (44). The ratio of *Firmicutes* to *Bacteroidetes* is widely accepted to play an important role in maintaining normal intestinal homeostasis, and the dysbiosis of this ratio can lead to some diseases (45, 46). In some diarrhea-related diseases, *Bacteroidetes* were lower, and *Firmicutes* were higher (47, 48). The *Firmicutes/Bacteroidetes* ratio was also significantly increased in calves with DHD, which reflected the disorder of the gut microbiota. At the genus level, *Escherichia-Shigella* and *Enterococcus* were remarkably higher in DHD calves. *Escherichia-Shigella* was the leading agent in the causes of diarrhea (49, 50). Increased *Enterococcus* has also been associated with some diarrhea-related diseases (51). We also detected a significant decrease in *Bacteroides, Subdoligranulum, Ruminococcus_2*, and *Sutterella* in fecal samples. *Bacteroides* have been found to be negatively correlated with colonic proinflammatory cytokines such as IL-6, IL-1β, and TNF-α (52), and *Sutterella* was negatively associated with inflammation (53). Thus, we speculated that an increased inflammatory response occurred in calves with DHD. *Lachnoclostridium* is associated with the production of short-chain fatty acids in the gut (54), and its significant decrease in DHD calves may be related to the significant changes in unsaturated fatty acid metabolism pathways. *Subdoligranulum* has also been found to be significantly decreased in a lot of intestinal diseases (55, 56). In this study, the correlation network analysis showed that DHD was associated with changes in the relevant structure and possible interaction structure of the intestinal microbiota. A similar disruption of the intestinal microbiota interaction network has been observed in other diseases presenting with diarrhea (57, 58). However, further studies are needed to determine the real role of intestinal microbiota interaction networks in the development of DHD.

Plasma metabolic profiles of DHD and healthy calves were significantly different. A total of 194 metabolites in plasma were identified as significantly different between the two groups (VIP > 2 and *P* < 0.05), which could serve as potential biomarkers of DHD in calves. Meanwhile, eight metabolic pathways were found to be associated with these differential metabolites, including pathways of glycerophospholipids, arachidonic acid, linoleic acid, and alpha-linolenic acid. Some of these metabolic pathways have also been shown to be the major disordered pathways in serum of animals with DHD (9, 59). Glycerophospholipids, the major components of cell membranes, alleviated diarrhea-related diseases (49, 60). Arachidonic acid, an important unsaturated fatty acid, is associated with many diseases including DHD (9, 61, 62). Linoleic acid is a necessary fatty acid that has been reported to be helpful in relieving diarrhea (63). It was reported that the increasing intake of essential fatty acids such as linoleic acid and alpha-linolenic acid improved immune status (64). Although some metabolites may be related to the pathogenesis of DHD, these metabolites still need to be quantitatively analyzed through targeted metabolomics, and the role of these metabolites in DHD pathogenesis should also be determined in future study.

Recently, a large number of studies have shown that metabolic changes and intestinal microbiota disorders were parallel during the disease progression (65, 66). Dysbiosis of the gut microbiota was accompanied with the alteration of plasma metabolome in calves with DHD. Spearman correlation analysis was performed to explore the correlation between the changes in fecal microorganisms and the plasma metabolites, and the results suggested that intestinal flora disturbance is related to metabolic phenotypic changes. *Escherichia-Shigella* was significantly correlated with metabolites involved in metabolism of arachidonic acid and alpha-linolenic acid, such as phosphatidylcholine and lecithin. *Fournierella* and *Ruminiclostridium_9* were significantly correlated with L-glutamate and sphingomyelin which are related to arginine, and proline metabolism, and the sphingolipid signaling pathway. These metabolites can also be produced by gut microbiota. In diarrhea cases, the disturbances in the metabolism of amino acids and glucose, and disorders of intestinal microecology were often observed (16, 67, 68). These studies also implied that the fecal microbes in DHD calves are closely related to the metabolic phenotype of the host.

Our results should be considered in the context of several constraints. First, the animal sample sizes were small, and larger cohorts need to be evaluated in future studies. Second, we did not verify the biomarkers obtained by other methods, and future studies should assess longitudinal microbiota before and during the onset of DHD, and expand analyses such as host responses and transcriptomics to more fully understand the pathogenesis of DHD, with a view of identifying targets for drug development.

## Conclusion

DHD disturbed the composition of fecal microbiota and plasma metabolites. The changes in the abundance of fecal microorganisms, especially *Escherichia-Shigella, Fournierella*, and *Ruminococcus_2*, and the concentrations of plasma metabolites, especially lecithin, phosphatidylcholine, and choline phosphatid, might affect the progression of DHD by interfering with metabolism of arachidonic acid, alpha-linolenic acid, linoleic acid, and glycerophospholipids. These significantly altered metabolic pathways and microorganisms may serve as diagnostic markers and potential therapeutic targets for DHD in calves. This finding provided a new insight for further investigating the mechanism of this kind of disease in calves.

## Data Availability Statement

The datasets presented in this study can be found in online repositories. The names of the repository/repositories and accession number(s) can be found in the article/Supplementary Material.

## Ethics Statement

The animal study was reviewed and approved by Laboratory Animal Ethics Commission of Lanzhou Institute of Husbandry and Pharmaceutical Sciences, CAAS.

## Author Contributions

JL and XS conceived and designed the work. JL coordinated technical support and funding. ZY wrote the manuscript. ZY, KanZ, KaiZ, GW, LW, JZ, ZQ, and ZG performed the experiments and collected the samples. JL and KaiZ reviewed the manuscript. All authors have read and approved to the published version of the manuscript.

## Funding

This work was financially supported by China Agriculture Research System of MOF and MARA, the National key research and development program of China (2017YFE0114400 and 2018YFD0502403), and special Fund of Chinese Central Government for Basic Scientific Research Operations in Commonweal Research Institutes (1610032021002).

## Conflict of Interest

The authors declare that the research was conducted in the absence of any commercial or financial relationships that could be construed as a potential conflict of interest.

## Publisher's Note

All claims expressed in this article are solely those of the authors and do not necessarily represent those of their affiliated organizations, or those of the publisher, the editors and the reviewers. Any product that may be evaluated in this article, or claim that may be made by its manufacturer, is not guaranteed or endorsed by the publisher.
