# Supplementary material for: Integrated 16S rDNA Gene Sequencing and Untargeted Metabolomics Analyses to Investigate the Gut Microbial Composition and Plasma Metabolic Phenotype in Calves With Dampness-Heat Diarrhea
Source: Front Vet Sci. 2022 Feb 15;9:703051. doi: 10.3389/fvets.2022.703051 (PMC8885629; doi:10.3389/fvets.2022.703051)
Supplement: Supplementary file 1 [file Data_Sheet_1.zip › Supplementary materials.docx]

**Supplementary Material and Methods**

**1 16S rRNA bioinformatics analysis**

1.1 Quality control and reads assembly

1.1.1Reads filtering

Raw data containing adapters or low quality reads would affect the following assembly and

analysis. Thus, to get high quality clean reads, raw reads were further filtered according to the

following rules using FASTP (Chen et al., 2018) (version 0.18.0):

1) Removing reads containing more than 10% of unknown nucleotides (N);

2) Removing reads containing less than 50% of bases with quality (Q-value)＞20.

1.1.2 Reads assembly

Paired end clean reads were merged as raw tags using FLSAH (Magoc and Salzberg, 2011) (version 1.2.11) with a minimum overlap of 10bp and mismatch error rates of 2%.

1.1.3 Raw tag filtering

Noisy sequences of raw tags were filtered by QIIME(Caporaso et al., 2010) (version 1.9.1 ) pipeline under specific filtering conditions(Bokulich et al., 2013) to obtain the high-quality clean tags. The filtering conditions are as follows：

1)Break raw tags from the first low quality base site where the number of bases in the continuous

low quality value (the default quality threshold is <=3) reaches the set length (the default length is

3)；

2)Then, filter tags whose continuous high-quality base length is less than 75% of the tag length.

1.1.4 Chimera checking and removal (Only for 16S sequencing analysis)

Clean tags were searched against the reference database (version r20110519, http://drive5.com/uchime/uchime_download.html) to perform reference-based chimera checking

using UCHIME algorithm(Edgar et al., 2011). All chimeric tags were removed and finally obtained effective tags were used for further analysis.

**2 LC-MS/MS analysis**

LC-MS/MS analyses were performed using an UHPLC system (1290, Agilent Technologies) with a UPLC HSS T3 column (2.1 mm × 100 mm, 1.8 μm) coupled to Q Exactive (Orbitrap MS, Thermo). The mobile phase A was 0.1% formic acid in water for positive, and 5 mmol/L ammonium acetate in water for negative, and the mobile phase B was acetonitrile. The elution gradient was set as follows: 0 min, 1% B; 1 min, 1% B; 8 min, 99% B; 10 min, 99% B; 10.1 min, 1% B; 12 min, 1% B. The flow rate was 0.5 mL/min. The injection volume was 2 μL. The QE mass spectrometer was used for its ability to acquire MS/MS spectra on an information-dependent basis (IDA) during an LC/MS experiment. In this mode, the acquisition software (Xcalibur 4.0.27, Thermo) continuously evaluates the full scan survey MS data as it collects and triggers the acquisition of MS/MS spectra depending on preselected criteria. ESI source conditions were set as following: Sheath gas flow rate as 45 Arb, Aux gas flow rate as 15Arb, Capillary temperature 320 oC, Full ms resolution as 70000, MS/MS resolution as 17500, Collision energy as 20/40/60 eV in NCE model, Spray Voltage as 3.8 kV (positive) or -3.1 kV (negative), respectively.

Reference

Bokulich, N.A., Subramanian, S., Faith, J.J., Gevers, D., Gordon, J.I., Knight, R., Mills, D.A., and Caporaso, J.G. (2013). Quality-filtering vastly improves diversity estimates from Illumina amplicon sequencing. *Nature Methods* 10**,** 884-890.

Caporaso, J.G., Kuczynski, J., Stombaugh, J., Bittinger, K., Bushman, F.D., Costello, E.K., Fierer, N., Pena, A.G., Goodrich, J.K., Gordon, J.I., Huttley, G.A., Kelley, S.T., Knights, D., Koenig, J.E., Ley, R.E., Lozupone, C.A., Mcdonald, D., Muegge, B.D., Pirrung, M., Reeder, J., Sevinsky, J.R., Tumbaugh, P.J., Walters, W.A., Widmann, J., Yatsunenko, T., Zaneveld, J., and Knight, R. (2010). QIIME allows analysis of high-throughput community sequencing data. *Nature Methods* 7**,** 335-336.

Chen, S., Zhou, Y., Chen, Y., and Gu, J. (2018). fastp: an ultra-fast all-in-one FASTQ preprocessor. *Bioinformatics* 34**,** 884-890.

Edgar, R.C., Haas, B.J., Clemente, J.C., Quince, C., and Knight, R. (2011). UCHIME improves sensitivity and speed of chimera detection. *Bioinformatics* 27**,** 2194-2200.

Magoc, T., and Salzberg, S.L. (2011). FLASH: fast length adjustment of short reads to improve genome assemblies. *Bioinformatics* 27**,** 2957-2963.
